# Motor Contributions to the Perception of Relative Phase

**DOI:** 10.1037/a0037351

**Published:** 2014-07-07

**Authors:** Richard Cook, Anne Gaule, Clarisse Aichelburg, Clare Press

**Affiliations:** 1 Department of Psychology, City University London; 2 Division of Psychology and Language Sciences, University College London; 3 Institute of Cognitive Neuroscience, University College London; 4 Department of Psychological Sciences, Birkbeck, University of London

**Keywords:** action perception, mirror mechanism, motion perception, motor system, relative phase

## Abstract

The extent to which different stimulus elements move together, namely their *relative phase*, is a central visual feature of many social and physical systems; characterizing everything from the oscillations of a walker’s limbs to the alternating lights at pedestrian crossings. The experiments described here provide the first evidence of a motor contribution to the representation of relative phase. Using an interference paradigm, we demonstrate that a motor load dramatically impairs discrimination of relative phase. Comparable interference effects were observed for biological and mechanical stimuli, indicative of a domain-general mechanism. In addition, we show that the same motor load has little effect on a similar static-angle matching task, and that an auditory rhythmic load did not interfere with phase discriminations in the same way as the motor load. These results suggest that the motor system contributes to the perception of relative phase; information crucial for interpreting our social and physical environments.

The discovery of mirror neurons, responsive both during action observation and execution (Rizzolatti & Craighero, 2004), prompted several action-specific motor theories of perception. These argue that internal simulation, mediated by a putative mirror neuron system, contributes to the visual perception of actions (e.g., Schütz-Bosbach & Prinz, 2007; Wilson & Knoblich, 2005). Consistent with action-specific motor theories, the performance of concurrent motor tasks modulates action perception, indicative of a causal motor contribution to perception (e.g., Christensen, Ilg, & Giese, 2011; Jacobs & Shiffrar, 2005). For example, when point light displays of arm movements are aligned in space and time with participants’ own actions, perception is enhanced relative to baseline, whereas spatial or temporal distortions impair perception (Christensen et al., 2011).

Discussion of action-specific motor theories has overshadowed evidence that the motor system also contributes to the perception of static (James & Gauthier, 2009; Müsseler & Hommel, 1997) and dynamic (Hu & Knill, 2010; Wohlschläger, 2000) stimuli that possess neither the biological form nor kinematic signature of actions. For example, preparation of right- or left-sided actions impairs detection of backward-masked schematic arrows pointing toward the same side of space (Müsseler & Hommel, 1997). Similarly, rapidly alternating lights arranged in a circular formation give rise to bi-stable apparent motion, perceived as either clockwise or counterclockwise rotation, but performing concurrent clockwise or counterclockwise actions promotes perception of rotation in the compatible direction (Wohlschläger, 2000). Given the nature of the stimuli used, these effects do not appear to be mediated by mechanisms recruited specifically when perceiving biological form or kinematics (Schubotz, 2007; see also Fadiga, Fogassi, Gallese, & Rizzolatti, 2000, for discussion of common neural populations recruited by action performance and observation of nonaction stimuli).

The present experiments extend our knowledge of domain-general motor contributions to perception, by demonstrating that motor processes contribute to the representation of relative phase (hereafter *phase*; the extent to which different stimulus elements move together) and moreover, do so equally for biological and mechanical stimuli. Representing phase accurately is crucial for perceiving many actions, notably running and walking, where an actor’s right and left limbs typically oscillate with a phase of 180° (they are always at opposite points in their cycle). However, phase is an important visual feature of many other biological and nonbiological systems; for example, the cardiac cycle of a beating heart^1^; the oscillations of an engine’s piston rods; the automated processes executed on assembly lines. Of note, hard-to-perform phase relationships, that is, those that are not 0° or 180°, are also hard-to-perceive (Bingham, Schmidt, & Zaal, 1999; Tuller & Kelso, 1989; Zaal, Bingham, & Schmidt, 2000), a correlation which raises the possibility that the motor system makes a causal contribution to the perception of phase.^2^ The present experiments addressed this hypothesis.

## Experiment 1

In Experiment 1 participants judged whether two sequentially presented phase relationships were, or were not, identical. Participants completed the phase-matching task both with and without a load placed on their motor system, induced by the performance of a concurrent finger-tapping task. If the motor system contributes to the perception of phase, a motor load should impair phase discrimination (e.g., Christensen et al., 2011).

### Method

Fourteen healthy adults (two males; *M*_age_ = 21.3 years, *SD*_age_ = 3.8 years; all right-handed) participated in the experiment. Three were replacements for participants who failed to execute the concurrent task at the appropriate frequency (having segmented block duration into consecutive 4-s windows, excluded participants were outside the desired 1.25:2 Hz range for more than 20% of the windows).

**Figure 1 fig1:**
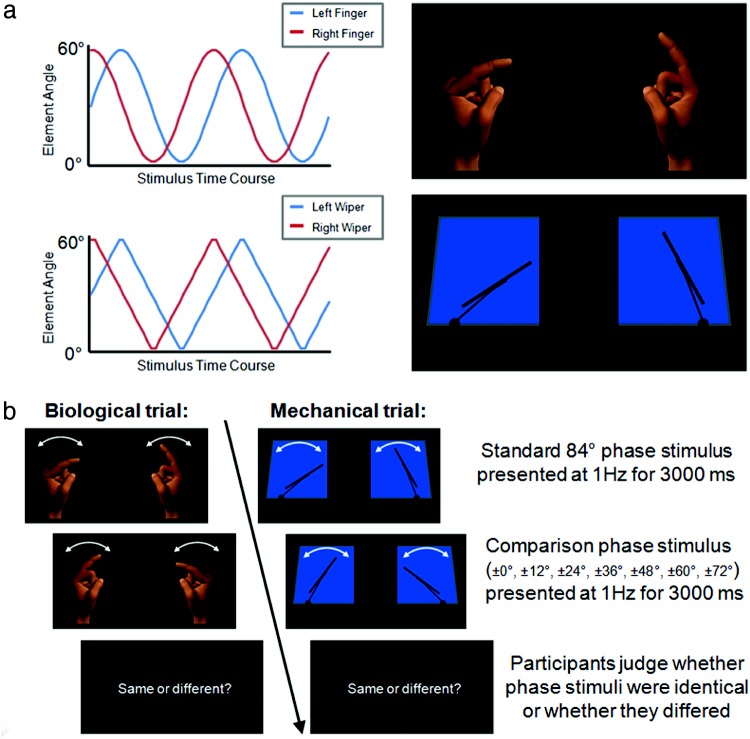
(a) Biological and mechanical phase stimuli were created by animating schematic depictions of windshield wiper-blades with constant velocity motion, characteristic of mechanically produced movement, and index-finger avatars with a sinusoidal velocity profile, which approximates human movement. The velocity profiles of the resultant biological and mechanical 84° phase stimuli are shown in the left panel. Biological and mechanical stimuli were produced and manipulated in e-Frontier Poser 7.0 and Microsoft PowerPoint. Hand contexts subtended 6° of visual angle horizontally and 13° vertically. Windshield contexts subtended 10° horizontally and 10° vertically. The center-to-center distance between the hands and windshields was 19°. (b) Illustration of the display sequence of dynamic phase discrimination trials employed in Experiment 1. Two phase stimuli were presented sequentially for 3,000 ms each with an inter-stimulus-interval of 750 ms. Participants judged whether the phase of the second stimulus was identical to that of the first or whether it differed. The color version of this figure appears in the online article only.

Stimuli were created by animating schematic depictions of windshield wiper-blades with constant velocity motion, characteristic of mechanically produced movement, and human index-finger avatars with a sinusoidal velocity profile, which better approximates human “minimum-jerk” movement (Figure 1a). Within each cycle (always of 1,000 ms duration/1 Hz), left and right stimulus elements (either fingers or wiper-blades) completed arcing trajectories from the 0° vertical position to a 60° angle toward the display center, and back again. The finger or wiper movements were always out of phase. Thirteen wiper- and 13 finger-stimuli presented left and right elements with systematically varying phase relationships (out-of-phase from 12° to 156° in increments of 12°). Movie stimuli (30 frames-per-second audio-visual-interleaved files) were presented on a CRT monitor (85 Hz refresh rate). Experimental programs were written in Matlab with Psychtoolbox (Brainard, 1997; Pelli, 1997).[Fig-anchor fig1]

The concurrent task required participants to tap on a peripheral keypad at a frequency of between 1.25 Hz and 2 Hz, with their left hand. If their tapping rate was too slow or too fast, low- and high-pitch tones sounded for 500 ms. The tapping frequency was greater than the frequency of the biological and mechanical phase stimuli, ensuring that the motor system was loaded with dynamic information different from that presented visually. Participants’ hands and the keypad were occluded from view.

Trials presented two phase stimuli sequentially, each for 3,000 ms, with an inter-stimulus-interval (ISI) of 750 ms, and an intertrial interval (ITI) of 1,200 ms. Participants continued to perform the concurrent tapping task during both the ISI and ITI. Participants judged whether the phase of the second stimulus was identical, or not, to that of the first (Figure 1b). Participants recorded their judgments with a mouse click made with their right hand. The first stimulus (“the standard”) always depicted a phase relationship of 84°. The second stimulus (“the comparison”) could either be identical (a phase difference of 0°) or differ by ± 12°, ± 24°, ± 36°, ± 48°, ± 60°, or ± 72°. The experiment comprised two blocks of 70 trials; one conducted with the motor load, one without. Block order was counterbalanced. Finger and wiper trials were interleaved randomly. Each of the seven interstimulus phase differences appeared an equal number of times within blocks. Blocks began with five practice trials.

Separate *d*-prime statistics estimated participants’ ability to detect each level of interstimulus phase difference, with and without the additional motor load (Macmillan & Creelman, 1991). Hits were correct “different” responses in the presence of an interstimulus difference. False alarms were “different” responses in the presence of a 0° interstimulus phase difference. Significance testing was conducted on the *d*-prime distributions.

### Results and Discussion

**Figure 2 fig2:**
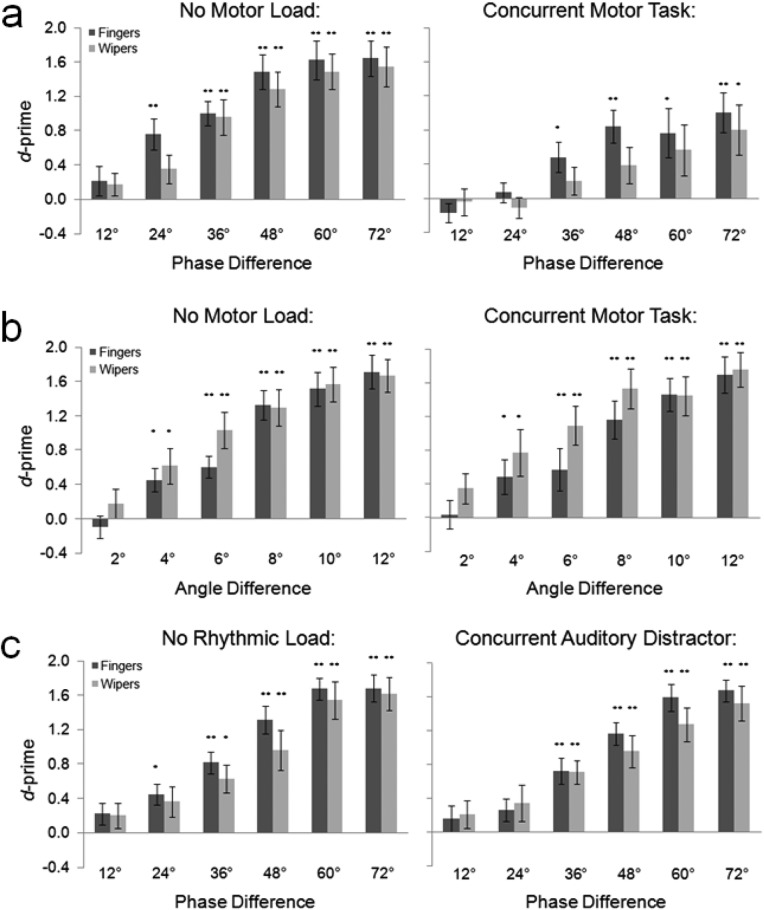
(a) Results of Experiment 1. The presence of a motor load, induced by the performance of a concurrent finger-tapping task, impaired participants’ ability to discriminate biological and mechanical phase relationships. (b) Results of Experiment 2. The presence of the same motor load had no effect on participants’ ability to match static configurations of biological and mechanical stimuli. (c) Results of Experiment 3. The presence of a rhythmic load, induced by a concurrent auditory distractor task had no effect on participants’ ability to discriminate biological and mechanical phase relationships. In all analyses, *d*-primes of zero indicate chance levels of discrimination. One-sample *t* tests were used to test whether distributions exceeded chance levels of discrimination (* denotes two-tailed significance at *p* < .05; ** denotes two-tailed significance at *p* < .001). Contrasts marked ** survive correction for multiple comparisons.

The discrimination data (Figure 2a) were subjected to ANOVA with motor load (concurrent task, no concurrent task), stimulus type (biological, mechanical), and interstimulus phase difference (12°, 24°, 36°, 48°, 60°, 72°) as within-subjects factors. The analysis revealed significant main effects of phase difference [*F*(5, 65) = 19.155, *p* < .001, η^2^ = .60] and motor load [*F*(1, 13) = 17.608, *p* < .001, η^2^ = .58], indicating that detection was better for large phase differences, and in the absence of the motor load. A phase × motor load interaction was also observed [*F*(5, 65) = 3.558, *p* < .025, η^2^ = .22], whereby the presence of the motor load disproportionately impaired detection of smaller phase differences. Importantly, there was no sign of a motor × stimulus (*p* > .8), or motor × stimulus × phase (*p* > .48) interaction, indicating that the motor task interfered equally when judging biological and mechanical phase relationships. There were no other main effects or interactions (all *p*s > .09). Performance of the concurrent task dramatically impaired participants’ ability to detect phase differences, indicative of a causal motor contribution to perception. The comparable interference observed for biological and mechanical stimuli suggests that this contribution is mediated by a domain-general mechanism; one not specifically tuned to the form or kinematics of actions (Schubotz, 2007).[Fig-anchor fig2]

## Experiment 2

Experiment 2 sought to determine whether the motor load in Experiment 1 interfered with encoding of spatiotemporal features of the phase stimuli or their simple spatial features. If the motor load interfered with representation of simple spatial features, it should also impair performance on a static angle-matching task of equivalent difficulty. Participants therefore judged whether sequentially presented configurations of static fingers or wiper-blades comprised the same angular difference, both with and without the motor load.

### Method

Fourteen healthy adults (10 males; *M*_age_ = 27.8 years, *SD*_age_ = 7.2 years; all right-handed) participated in the experiment. Two were replacements for participants who failed to execute the concurrent task at the appropriate frequency (using the criterion adopted in Experiment 1).

The stimuli were 52 static configurations of fingers and wiper-blades similar to those depicted in the dynamic stimuli used in Experiment 1. Half presented the left element angled 20° toward the display center. The angle of the right element was varied incrementally from 30° to 54° in steps of 2° yielding 13 stimuli within each set. The remaining stimuli were produced using the complementary manipulation, whereby the right element was held constant at 20° and the left element varied systematically. These angular differences were chosen on the basis of piloting to equate approximately *d*-primes with Experiment 1.

**Figure 3 fig3:**
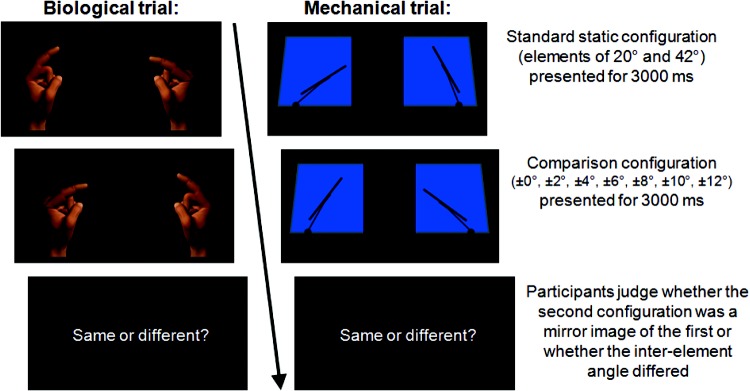
Illustration of the display sequence of the static angle-matching trials employed in Experiment 2. Two stimuli were presented for 3,000 ms each with an inter-stimulus-interval of 750 ms. Participants judged whether or not the second configuration was a mirror image of the first. The color version of this figure appears in the online article only.

Each trial presented two static configurations sequentially (either wipers or fingers) for 3,000 ms with an ISI of 750 ms (see Figure 3). Participants judged whether the second configuration was an exact mirror image of the first.^3^ The first configuration comprised elements of 20° and 42°. The second configuration could either be an exact mirror image or deviate by ± 2:12°. Seven interconfiguration differences each appeared an equal number of times.[Fig-anchor fig3]

### Results and Discussion

The discrimination data (Figure 2b) were subjected to ANOVA with motor load (concurrent task, no concurrent task), stimulus type (biological, mechanical), and interconfiguration difference (12°, 24°, 36°, 48°, 60°, 72°) as within-subjects factors. The analysis revealed a significant main effect of interconfiguration difference [*F*(5, 65) = 64.746, *p* < .001, η^2^ = .83], but no main effect of motor load (*p* > .70) or stimulus type (*p* > .20). No interactions were observed (all *p*s > .20). Comparison of discrimination performance across the dynamic (Experiment 1) and static (Experiment 2) tasks, in the absence of motor load, confirmed that the tasks were equated for difficulty (*p* > .70). Crucially, there was a significant task × motor load interaction [*F*(1, 26) = 11.805. *p* < .01, η^2^ = .31], indicative of disproportionate motor interference in the dynamic task. Despite dramatically impairing the discrimination of phase, the motor load failed to impair performance on a static angle-matching task. The effect observed in Experiment 1 is therefore unlikely to reflect interference with representation of simple spatial features.

## Experiment 3

Experiment 3 sought to determine whether the interference effect observed in Experiment 1 was due to a rhythmic load, induced by asynchronous afferent sensation. For example, the auditory and proprioceptive consequences of the tapping movements were asynchronous with the phase stimuli, and may have detracted from perception. To test whether the presence of asynchronous sensation can elicit a similar performance decrement to that seen in Experiment 1, participants made phase discriminations, either with no distraction, or while trying to detect rate changes in a stream of auditory tones. If the effect seen in Experiment 1 was due to additional rhythmic load, similar interference should be observed.

### Method

Fourteen healthy adults (10 males; *M*_age_ = 23.9 years, *SD*_age_ = 4.6 years; all right-handed) participated in the experiment. The distractor task required participants to listen to a continuous stream of 300 Hz tones. At random intervals the rate of tones slowed, from one every 800 ms, to one every 1,500 ms, for a period of 7,500 ms. When participants detected a rate change, they made a keypress response. The baseline rate was selected to be equivalent to the tapping frequency executed by participants in Experiment 1 and 2. Participants’ hands and the keypad were occluded from view. The discrimination task was identical to that employed in Experiment 1. Participants completed the concurrent task well, detecting 97.6% of the rate changes. This performance was comparable to that of the motor task used in Experiments 1 and 2 where appropriate tapping frequency (defined above) was seen in 95.8% and 95.5% of the 4-s intervals, respectively.

### Results and Discussion

The discrimination data (Figure 2c) were subjected to ANOVA with rhythmic load (present, absent), stimulus type (biological, mechanical), and phase difference (12°, 24°, 36°, 48°, 60°, 72°) as within-subjects factors. The analysis revealed a significant main effect of phase [*F*(5, 65) = 46.912, *p* < .001, η^2^ = .78], but no effect of stimulus type (*p* > .15) or rhythmic load (*p* > .50). No interactions were observed (all *p*s > .40). Comparison of phase discrimination across Experiments 1 and 3 revealed no significant difference between the groups in the absence of load (*p* > .55) indicating that they were matched in their baseline discrimination ability. Crucially, a significant load (present, absent) × distractor (motor, rhythmic) interaction was observed [*F*(1, 26) = 7.684. *p* < .01, η^2^ = .23], indicative of disproportionate interference induced by the presence of a motor load. The fact that phase discrimination was unaffected by the auditory rhythmic load, suggests a) that the interference effect seen in Experiment 1 was not caused by asynchronous auditory feedback from the tapping task; and b) that alternative forms of distraction do not necessarily impair phase discrimination.

## General Discussion

Representing phase accurately is crucial for interaction with our social and physical environments. However, despite its significance, relatively little is known about the perceptual representation of phase. Together, the present findings suggest that the motor systems responsible for planning and producing actions, contribute to the perception of phase relationships, both for biological and mechanical stimuli. The suggestion that phase perception recruits the motor system is consistent with previous findings that hard-to-perform phase relationships are also hard to perceive (Bingham et al., 1999; Zaal et al., 2000). Moreover, this conclusion accords with evidence that perception of walking and running—actions defined by prominent phase relationships—is impaired by concurrent action performance (e.g., Jacobs & Shiffrar, 2005) and by the application of disruptive transcranial magnetic stimulation (van Kemenade, Muggleton, Walsh, & Saygin, 2012) or neurological lesions (Saygin, 2007), to regions of premotor cortex.

How did the motor component of the load impair phase judgments? To represent the phase stimuli, participants needed to integrate changes in each element’s spatial location over time. Therefore, one possibility is that the concurrent tapping task and the trajectories of stimulus elements excited competing spatiotemporal codes, impairing phase judgments. Alternatively, the motor task may have interfered at a purely temporal level, due to differences between the observed and executed frequencies. This interpretation would be consistent with findings that cortical and subcortical motor structures are recruited when judging temporal features, including duration (Coull, Nazarian, & Vidal, 2008; Ivry & Schlerf, 2008; Schubotz, Friederici, & von Cramon, 2000). Participants may have found it harder to judge the intervals between the zenith of the left and right stimulus elements, because they were asynchronous with the tapping movements.

The present results strengthen the view that motor structures help us perceive a range of stimuli extending beyond the actions of conspecifics (Schubotz, 2007; Schubotz & von Cramon, 2004). These findings extend this line of research, by showing that the motor system supports the spatiotemporal representation of mechanical phase relationships, and that this contribution is equivalent to that seen with actions. Motor contributions to the perception of phase thus seem unlikely to reflect the operation of mechanisms tuned to biological form or kinematics.

## Footnotes

1 For example, a heart surgeon might observe the left atrium and aorta contracting in antiphase. We note that humans do not have x-ray vision.

2 While the finding that hard-to-perform actions are hard to perceive is consistent with a motor contribution to perception, we wish to clarify that this is not the interpretation offered by the authors.

3 This task therefore required participants to judge whether the first image could be replicated exactly by subjecting the second image to reversal about the vertical midline.
